# Supplementary material for: Conditional chemoconnectomics (cCCTomics) as a strategy for efficient and conditional targeting of chemical transmission
Source: eLife. 2024 Apr 30;12:RP91927. doi: 10.7554/eLife.91927 (PMC11060718; doi:10.7554/eLife.91927)
Supplement: Figure 3—source data 3. [file elife-91927-fig3-data3.zip › Figure 3 source data3 part 1.zip!/Figure 3-Source data 3-part1/1071f2-1-CRISPResso_Report_ztwktvfp.zip!/CRISPResso_on_1071f2_2/1a.Read_barplot.pdf]

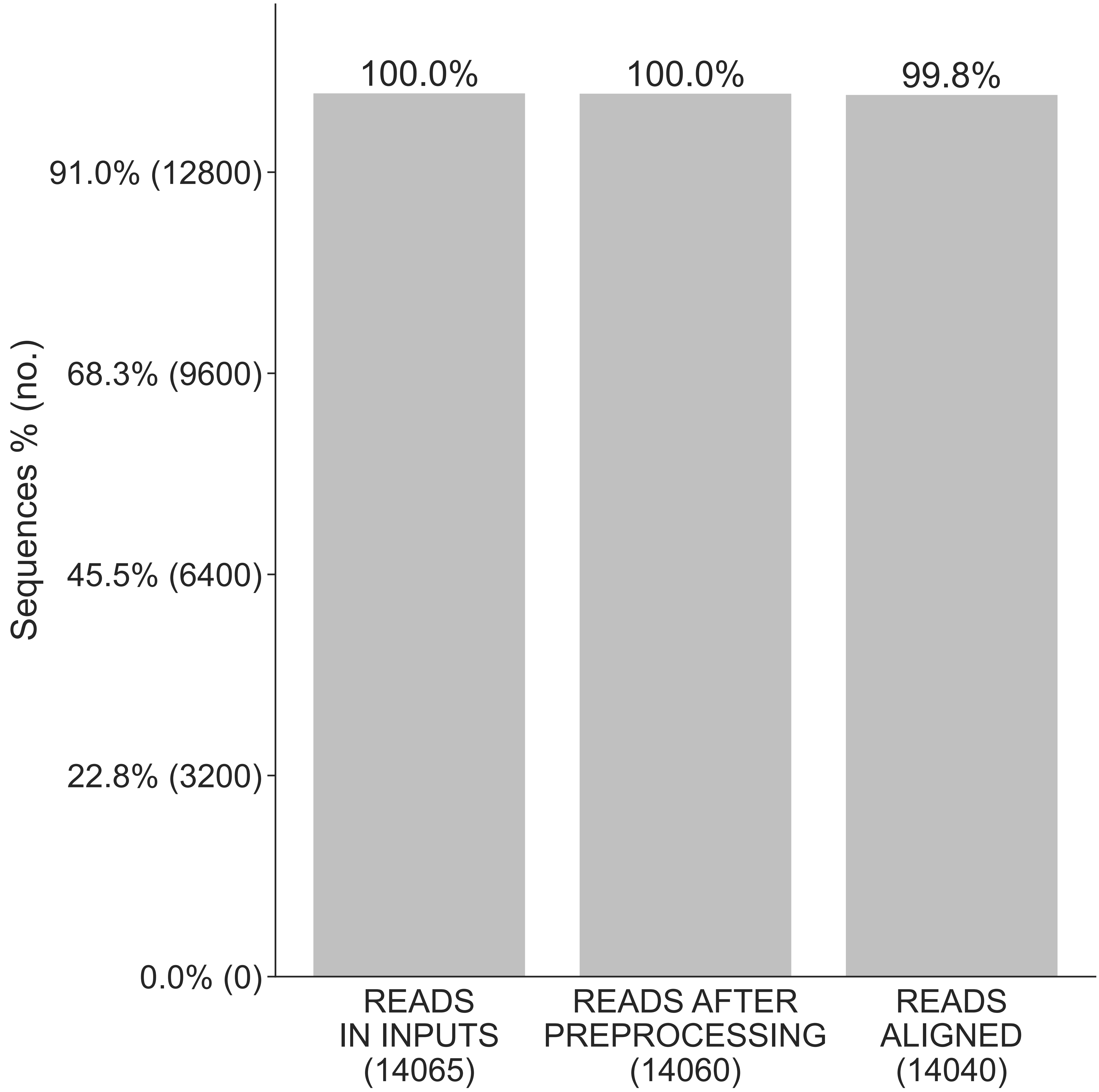
100.0%
100.0%
99.8%
Sequences % (no.)
91.0% (12800)
68.3% (9600)
45.5% (6400)
22.8% (3200)
0.0% (0)
READS IN INPUTS (14065)
READS AFTER PREPROCESSING (14060)
READS ALIGNED (14040)
